# Prediction of respiratory complications by quantifying lung contusion volume using chest computed tomography in patients with chest trauma

**DOI:** 10.1038/s41598-023-33275-z

**Published:** 2023-04-19

**Authors:** Na Hyeon Lee, Seon Hee Kim, Sang-hyup Seo, Byeong-Jun Kim, Chi-Seung Lee, Gil Hwan Kim, Sung Jin Park, Seon Hyun Kim, Dong Yeon Ryu, Ho Hyun Kim, Sang Bong Lee, Chan Ik Park, Jae Hun Kim

**Affiliations:** 1 Department of Trauma Surgery, School of Medicine, Pusan National University, Biomedical Research Institute, Pusan National University Hospital, 179 Gudeok-Ro, Seo-Gu, Busan, 49241 Republic of Korea; 2 Busan Center for Medical Mathematics, National Institute for Mathematical Sciences, Daejeon, Republic of Korea; 3 Department of Convergence Medicine and Biomedical Engineering, School of Medicine, Pusan National University, Biomedical Research Institute, Pusan National University Hospital, Busan, Republic of Korea

**Keywords:** Diseases, Risk factors, Signs and symptoms

## Abstract

Pulmonary contusion is an important risk factor for respiratory complications in trauma patients. Hence, we aimed to determine the relationship between the ratio of pulmonary contusion volume to the total lung volume and patient outcomes and the predictability of respiratory complications. We retrospectively included 73 patients with a pulmonary contusion on chest computed tomography (CT) from 800 patients with chest trauma admitted to our facility between January 2019 and January 2020. Chest injury severity was expressed as the ratio of pulmonary contusion volume to total lung volume by quantifying pulmonary contusion volume on chest CT. The cut-off value was 80%. Among the 73 patients with pulmonary contusion (77% males, mean age: 45.3 years), 28 patients had pneumonia, and five had acute respiratory distress syndrome. The number of patients in the severe risk group with > 20% of pulmonary contusion volume was 38, among whom 23 had pneumonia. For predicting pneumonia, the area under the receiver operating characteristic curves for the ratio of pulmonary contusion volume was 0.85 (95% confidence interval 0.76–0.95, p = 0.008); the optimal threshold was 70.4%. Quantifying pulmonary contusion volume using initial CT enables identifying patients with chest trauma at high risk of delayed respiratory complications.

## Introduction

Pulmonary contusion is an important risk factor for respiratory complications, including pneumonia and acute respiratory distress syndrome (ARDS), in trauma patients. In addition, pulmonary contusions, which account for approximately 25% of all deaths, are an independent risk factor for mortality^1^. A pulmonary contusion can occur due to any mechanism, such as crushing and penetrating injuries and blunt trauma. These mechanisms damage the alveolar capillaries, causing blood and other body fluids to accumulate in the lung tissue, thereby interfering with gas exchange and leading to hypoxia^2^. It is also related to the activation of inflammatory response via innate immunity following trauma^3^. Patients are treated with adequate oxygen supplementation, and mechanical ventilation may be required in severe cases. Pulmonary contusions usually resolve spontaneously with supportive care; however, they may progress to respiratory complications during treatment in intensive care units (ICUs).

The scoring systems for evaluating injury severity in patients with pulmonary contusions include the chest abbreviated injury scale (AIS) and injury severity score (ISS). However, because these scoring systems are obtained through anatomical abnormalities, they have limitations in representing functional impairment or predicting prognosis. Therefore, it is necessary to introduce a method for measuring the injury severity that reflects pulmonary function better. Hypoxemia is induced by ventilation-perfusion mismatch and an increase in shunt in areas with pulmonary contusion; hence, the wider the pulmonary contusion area, the more severe the degree of hypoxemia^2^. Pulmonary contusions may be an important risk factor for the progression of respiratory complications. Chest radiography has limitations in confirming the pulmonary contusion volume. However, it is easier to visualize pulmonary contusions and measure their area through chest computed tomography (CT). Contrast-enhanced CT is a good tool to identify bleeding and ground-glass opacity (GGO) due to parenchymal damage more clearly in chest CT. Recently, studies have been conducted using lung segmentation technology for chest CT. Ultimately, it is important to extract the total lung and contusion volumes using CT. Automated lung segmentation algorithms are limited in handling routine data because they are developed for limited datasets of specific diseases or cases. According to a study by Hofmanninger et al.^4^, models trained with routine data were more effective in lung segmentation than public datasets in generalizability to a wide spectrum of pathologies^4^. Including or excluding pathologies, such as effusion or pneumothorax, when using these trained models is a matter of definition and application. To determine the Hounsfield units (HU) range for segmenting the entire lung area and the area expected to have normal lung function in trauma patients, we used a three-dimensional (3D) slicer and hand labeling data as a reference.

Therefore, this study aimed to determine the relationship between patients and the ratio of the pulmonary contusion volume caused by all mechanisms of trauma to the total lung volume. We also aimed to determine whether respiratory complications can be predicted.

## Methods

Informed consent was obtained from all participants or their legal guardians. All methods were conducted in accordance with the relevant guidelines and regulations. This study was approved by the ethics committee of Pusan National University Hospital (IRB No. 2011-017-097, date of IRB approval: November 12, 2020).

### Study design and patients

**Figure 1:**
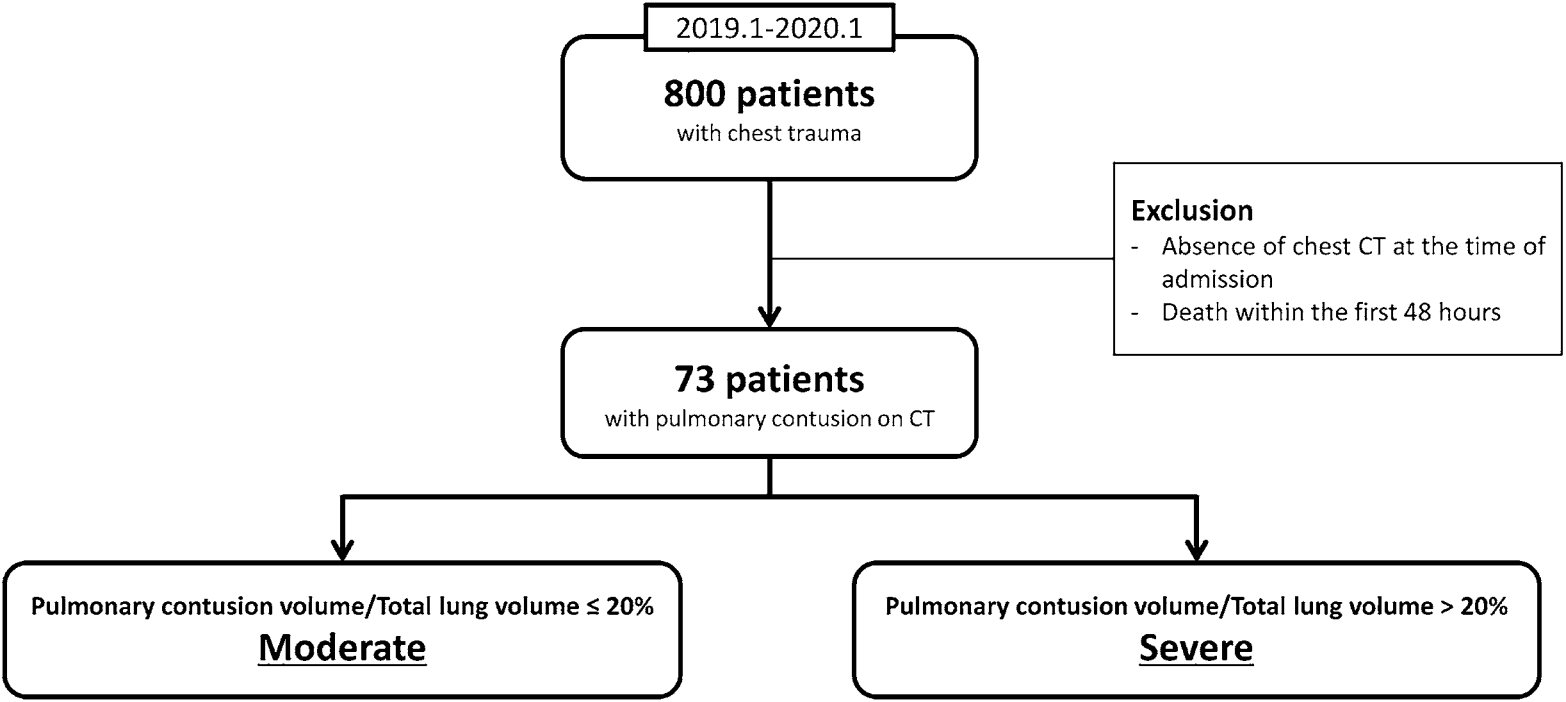
Study design flow chart. *CT* computed tomography.

Among 800 patients with chest trauma admitted to our level I trauma center between January 2019 and January 2020, we retrospectively analyzed 73 patients diagnosed with pulmonary contusion based on chest CT. The following patients were included: (1) Patients injured because of trauma, (2) those diagnosed with pulmonary contusion by chest CT, and (3) those with a chest AIS score of one or higher. The exclusion criteria were as follow: (1) absence of chest CT at the time of admission and (2) death within the first 48 h of admission (Fig. 1).

### Data collection

The data collected included patient demographics, mechanism of injury, injury severity, mechanical ventilator application period, length of hospital stay, length of ICU stay, pneumonia, and ARDS. Data were obtained through medical records. Injury severity included ISS, chest AIS, head and neck AIS, initial Glasgow coma scale (GCS) score, transfusion requirement in the emergency room, and initial lactate level. In addition, the partial pressure of oxygen in arterial blood (PaO_2_) and inspiratory fraction of oxygen (FiO_2_) were obtained from daily arterial blood gas analysis and ventilator settings to calculate the PaO_2_/FiO_2_ ratio.

### Lung area segmentation algorithm on chest CT

Two physicians from the Department of Trauma Surgery with over 5 years of experience in thoracic surgery participated in the study to confirm the presence of pulmonary contusions on chest CT images.

**Figure 2:**
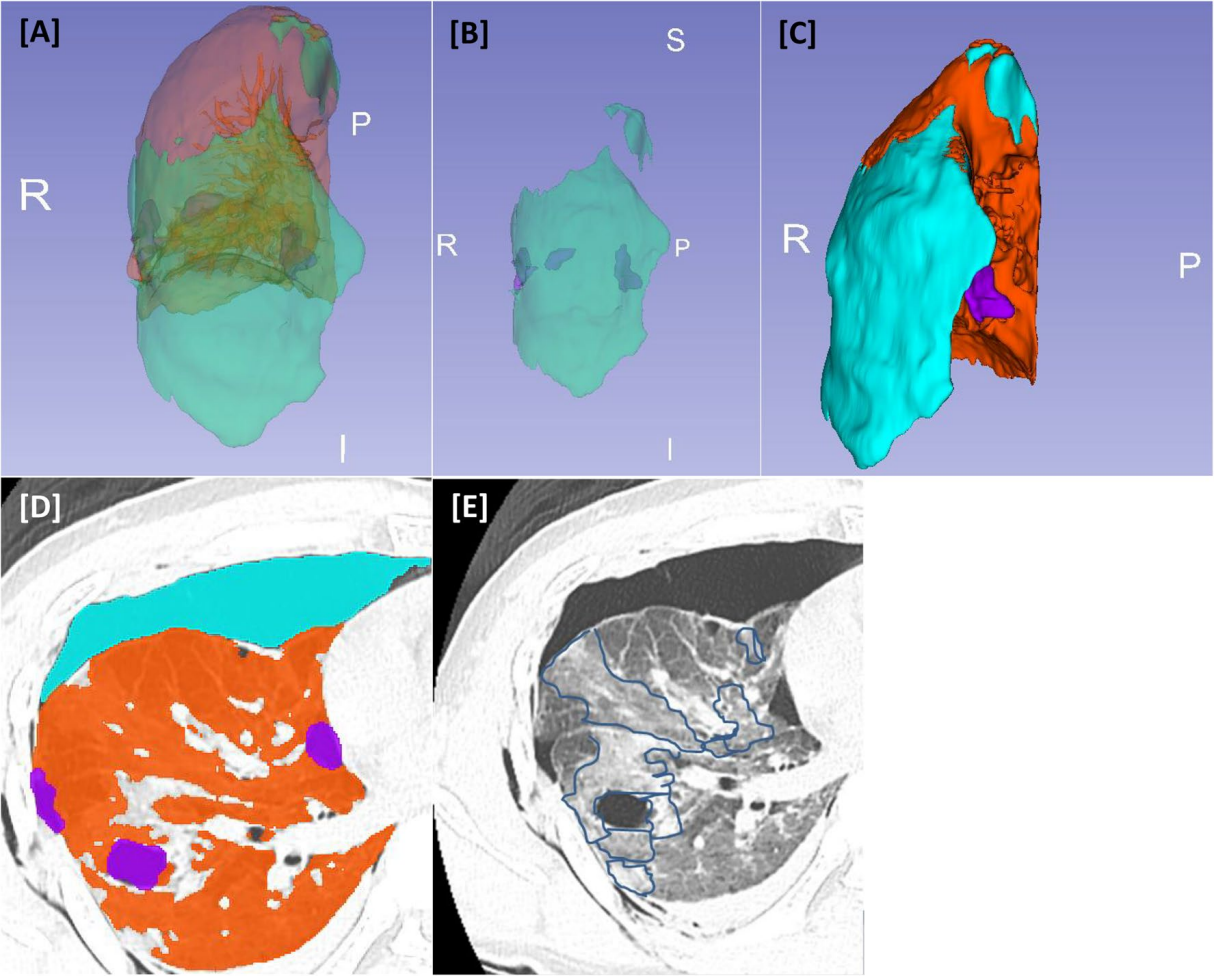
Process of lung segmentation with a three-dimensional (3D) slicer. The 3D model can be viewed after setting the most appropriate Hounsfield unit for lung segmentation. (**A**) anterior view, (**B**) parts excluding normal lung area, and (**C**) posterior view. Blue indicates the pneumothorax region, orange indicates the normal lung area, and purple indicates the segmented region with pulmonary contusion. (**D**) transverse view on chest computed tomography. (**E**) If there is an actual contusion and it is missing, it is indicated by hand labeling. *R* right, *P* posterior, *I* inferior.

Two methods of lung segmentation that complement each other were used. First, the total and normal lung volumes were obtained using 3D Slicer, which is a free, open-source software for medical image computing. After loading the CT Digital Imaging and Communications in Medicine images of the patients into 3D Slicer, we identified the HU range for the most appropriate lung segmentation. After adjusting the HU range, we compensated by hand labeling if a part with pulmonary contusion was missed (Fig. 2). The second method of lung segmentation involved a lung segmentation model created by introducing the *U*-net (R231) algorithm in Hofmanninger’s study^4^. The HU range, with reference to the first method, was found to range from − 950 to − 450, including the normal lung volumes that were not damaged except for pulmonary contusion in the lung segmentation model with the *U*-net (R231) algorithm. This algorithm utilized the *U*-net (R231CovidWeb) model, which additionally learns the lungs of coronavirus disease patients. We segmented the total, normal lung, and pulmonary contusion volume by contrasting these two methods. When the lungs were first segmented using this model, the normal lung volume was smaller than expected, as pneumothorax was excluded from the normal lung volume. Traumatic pneumothorax is often caused by laceration of the visceral pleura caused by rib fracture, alveolar rupture caused by increased alveolar pressure due to chest compression, or visceral or mediastinal pleura rupture, rather than due to underlying lung disease^5^. The collapsed lung was expected to expand after thoracostomy; hence, pneumothorax was included as the normal lung volume, not the pulmonary contusion.

### Three-dimensional filtering algorithm for pneumothorax detection

As previously described, a lung with pneumothorax can be visualized as a normal lung. This is because the air in pneumothorax has a different HU range from that in the alveoli; hence, additional search algorithms are needed to detect pneumothorax. Consequently, the search method was supplemented by applying the denosing technique for 3D filtering, which is an extension of two-dimensional filtering, to exclude the alveoli and only visualize the pneumothorax.

### Calculation of the ratio of the pulmonary contusion volume to the total lung volume

The ratio of the pulmonary contusion volume to the total lung volume was calculated by including the pneumothorax volume in the pulmonary contusion volume, using the results obtained from the search method. According to the results of previous studies, ARDS incidence sharply increases when the pulmonary contusion volume exceeds 20% of the total lung volume. Hence, when the pulmonary contusion volume was ≤ 20% of the total lung volume, it was defined as moderate, and when it was > 20%, it was defined as severe (Fig. 1)^1,6–8^.

### Primary outcome and definition of ARDS

Outcomes were obtained, including pneumonia, ARDS, in-hospital mortality, hospital stay, ICU stay, and ventilator application period. ARDS was defined as the absence of evidence of congestive heart failure with a PaO_2_/FiO_2_ ratio of ≤ 200 and bilateral diffuse infiltration on chest radiography.

### Statistical analysis

When appropriate, summary statistics are reported as median and interquartile range or mean with standard deviation. Categorical variables are expressed as numbers and percentages. The chi-square test and Fisher’s exact test were performed to compare the frequencies of categorical variables between the groups. The Mann–Whitney *U*-test and Wilcoxon rank-sum test were performed to compare the mean values of continuous variables. We used the receiver operating characteristic (ROC) curve and the area under the curve (AUC) to evaluate the predictive factors for pneumonia. In addition, a *t* test was performed for the GCS, ISS, and lactate levels, as well as the ratio of pulmonary contusion volume to total lung volume of the groups with and without pneumonia. Linear regression was performed to adjust for age. Statistical significance was set at a p-value of < 0.05. SPSS (version 22.0; IBM Corp., Armonk, NY, USA) was used to analyze the data.

## Results

### Patient characteristics

Among the 73 patients with pulmonary contusion identified by chest CT, 56 (77%) were males, and their mean age was 45.3 ± 15.9 years. The most common mechanisms of injury were motor vehicle crashes, followed by falls and crushing injuries, with 40 (54.8%), 26 (35.6%), and 5 (6.8%) cases, respectively. The mean ISS was 22.3 ± 8.9, and the mean chest AIS was 3.2 ± 0.7. The patients’ characteristics are summarized in Table 1. Twenty-three patients showed GGO-type pulmonary contusions, 30 patients had GGO and consolidations, and the remaining 20 patients had GGO, nodular opacity, and consolidations in non-segmental areas on chest CT images.

**Table 1:** Patient characteristics and in-hospital outcomes.

	Value (n = 73)
Demographics	
Age (mean ± SD)	45.3 ± 15.9
Male (%)	56 (76.7%)
Mechanism of injury (%)	
Motor vehicle crash	40 (54.8%)
Fall	26 (35.6%)
Crush injury	5 (6.8%)
Stab wound	1 (1.4%)
Assault	1 (1.4%)
Injury severity (mean ± SD)	
ISS	22.3 ± 8.9
Chest AIS	3.2 ± 0.7
Head and neck AIS	1.0 ± 1.5
GCS at ER	13.4 ± 3.1
Patients requiring blood transfusion	24
Physiologic parameters	
Lactate	3.8 ± 3.0
SBP	112.9 ± 31.6
Pulmonary status	
Pneumonia	28 (38.4%)
ARDS	5 (6.8%)
Ventilator days	7.4 ± 14.8
Patient outcomes	
ICU stay	9.4 ± 16.2
Hospital stay	31.9 ± 56.4
Mortality	2 (2.7%)

*ARDS* acute respiratory disease syndrome, *AIS* abbreviated injury scale, *ER* emergency room, *GCS* Glasgow coma scale, *ICU* intensive care unit, *ISS* injury severity score, *SBP* systolic blood pressure, *SD* standard deviation.

### In-hospital outcomes

**Figure 3:**
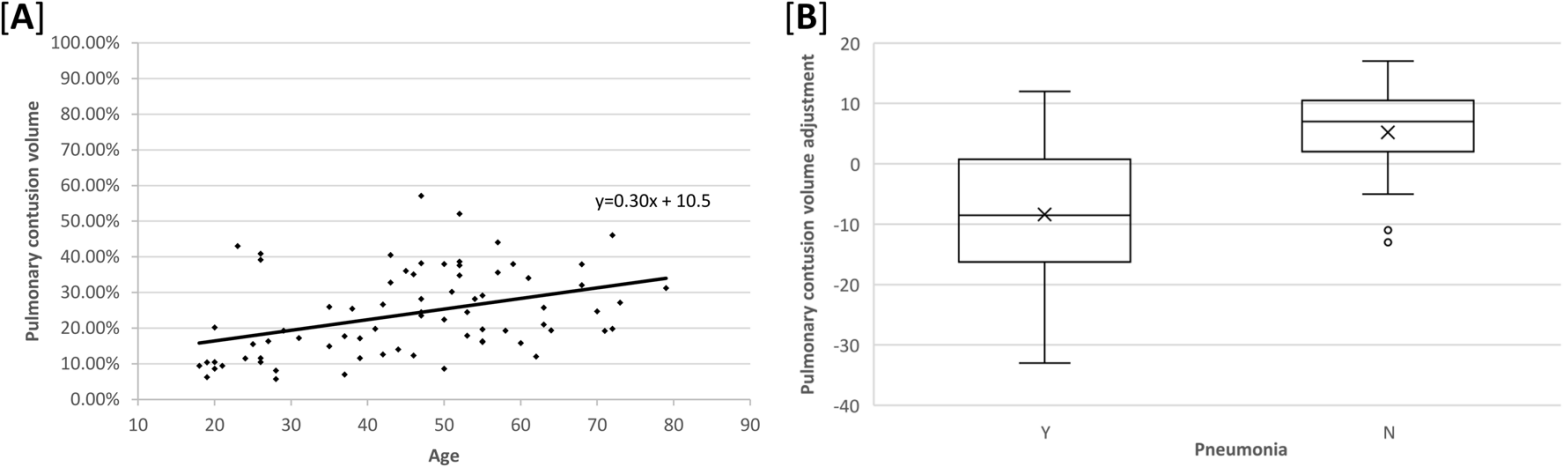
Age adjustment for pneumonia incidence according to pulmonary contusion volume. (**A**) Pneumonia incidence according to age has a regressive relationship. (**B**) In order to adjust for age, a simple linear regression is used to calculate the residual, and then the mean and standard deviation were calculated, and there was still a statistically significant difference between the moderate and severe groups (5.21 ± 7.12 and − 8.37 ± 11.21, respectively) (p < 0.001).

A total of 28 patients (38.4%) were diagnosed with pneumonia, five (6.8%) were diagnosed with ARDS, and two (2.7%) died during hospitalization. The number of patients with ≤ 20% of pulmonary contusion volume in the moderate-risk group was 35, while the number in the severe group was 38. The mean age was 39.3 ± 16.1 years in the moderate group and 50.9 ± 13.7 years in the severe group, showing a statistically significant difference with a p-value of ≤ 0.005. After adjusting for age, a statistically significant difference was observed in the occurrence of pneumonia between the moderate and severe groups (p < 0.0001) (Fig. 3). When injury severity was compared between the two groups, the severe group had a higher ISS; nonetheless, the results were not statistically significant (Table 1).

When the outcomes between the two groups were compared, pneumonia incidence was significantly higher in the severe group than that in the moderate group (five in the moderate group and 23 in the severe group). ARDS and in-hospital mortality were confirmed in five and two cases, respectively, in the severe group, but the difference in the incidence of these outcomes between the severe and moderate groups was not statistically significant (Table 2).

**Table 2:** Comparison between moderate and severe groups.

(Pulmonary contusion volume)/(total lung volume)	≤ 20% (moderate group)	> 20% (severe group)	p-value
N	35	38	
Demographics			
Age (mean ± SD)	39.3 ± 16.1	50.9 ± 13.7	< 0.005
Male	25	31	
Injury severity (mean ± SD)			
ISS	21.2 ± 7.7	23.4 ± 9.8	0.29
Chest AIS	2.8 ± 0.6	3.4 ± 0.7	0.07
GCS at ER	14.1 ± 2.5	12.8 ± 3.5	0.07
Patients requiring blood transfusion	7	17	0.07
Outcome			
Pneumonia	5	23	< 0.005
ARDS	0	5	0.090
Mortality	0	2	0.54
Hospital stay	35.2 ± 80.3	28.9 ± 20.2	0.66
ICU stay	6.5 ± 19.1	12.0 ± 12.9	0.17
Ventilator days	4.6 ± 17.3	9.9 ± 11.9	0.14

*ARDS* acute respiratory disease syndrome, *AIS* abbreviated injury scale, *ER* emergency room, *GCS* Glasgow coma scale, *ICU* intensive care unit, *ISS* injury severity score, *SD* standard deviation.

**Figure 4:**
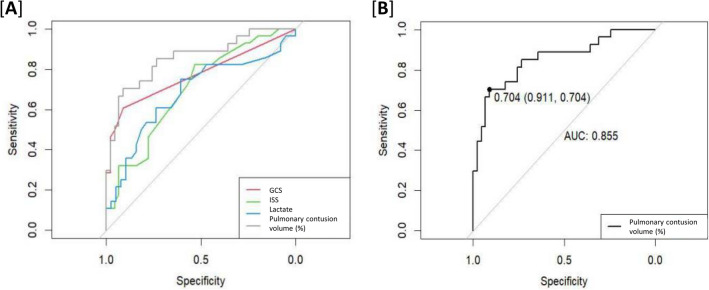
Receiver operating characteristic curve for the prediction of pneumonia.

Variables with an AUC exceeding 0.7 in the ROC curve for pneumonia prediction were the GCS score, ISS, lactate level, and percentage of pulmonary contusion volume (Fig. 4). The AUC was 0.77, 0.7, 0.73, and 0.85, respectively, showing the highest percentage of pulmonary contusion volume (95% confidence interval 0.76–0.95, p = 0.008, sensitivity 0.704, specificity 0.911). Additionally, the optimal threshold of the normal lung volume percentage for predicting pneumonia was 70.4%. The other optimal thresholds for GCS score, ISS, and lactate levels were 14.5, 19.5, and 2.45, respectively.

The *t* test performed for GCS score, ISS, lactate levels and the ratio of pulmonary contusion volume for the groups with and without pneumonia revealed that the GCS score was 11.3 ± 4.1 in the pneumonia group and 14.8 ± 1.0 in the non-pneumonia group (p = 0.0001), ISS was 26.0 ± 9.4 in the pneumonia group and 20.0 ± 7.8 in the non-pneumonia group (p = 0.0063), the lactate levels were 4.9 ± 3.6 in the pneumonia group and 3.0 ± 2.13 in the non-pneumonia group (p = 0.0207), and the ratio of pulmonary contusion volume was 33.04 ± 11.4 in the pneumonia group and 18.3 ± 8.4 (p < 0.0001) in the non-pneumonia group (Table 3).

**Table 3:** t-test for the pneumonia and non-pneumonia groups.

	Pneumonia	Non-pneumonia	p-value
N	28	45	
GCS (mean ± SD)	11.3 ± 4.1	14.8 ± 1.0	0.0001
ISS (mean ± SD)	26.0 ± 9.4	20.0 ± 7.8	0.0063
Lactate (mean ± SD)	4.9 ± 3.6	3.0 ± 2.1	0.0207
(Pulmonary contusion volume)/(total lung volume)	33.04 ± 11.4	18.3 ± 8.5	< 0.0001

*GCS* Glasgow coma scale, *ISS* injury severity score, *SD* standard deviation.

## Discussion

In trauma patients, lung contusions are a risk factor for respiratory complications^1–3,6,7^. Therefore, confirming the ratio of the pulmonary contusion volume to the total lung volume on chest CT at the initial stage of injury can help predict respiratory complications^5–7^. According to previous studies, pulmonary contusion can cause respiratory complications by cytokine secretion mechanism above the threshold, including ARDS and mechanical damage to the lungs. Therefore, it is important to identify the ratio of pulmonary contusion volume to the total lung volume using radiological tests.

This study analyzed two groups by dividing the participants into moderate and severe groups according to the ratio of pulmonary contusion volumes. Miller et al. reported that the risk of ARDS according to the ratio of the pulmonary contusion volume did not increase linearly, but increased rapidly when the contusion volume was 20% of the total volume^6^. Although the cause is unclear, it is presumed that there is a threshold for ARDS caused by an intrapulmonary shunt at the injury site^6^. Although we utilized a small sample size in this study, there was no statistically significant difference in injury severity, such as the number of patients requiring transfusion, ISS, chest AIS, and the GCS scores between the two groups. However, the ages of the patients in the moderate and severe groups showed statistically significant differences. As the initial pulmonary function test could not be performed in both groups, lung function could not be accurately compared. However, on chest CT, there were no significant differences in lung lesions that could impair lung function, such as pulmonary emphysema or interstitial lung disease. Depending on age, the damage to the lung itself seems to be different when impacted by trauma. As age increases, the composition of the components of each layer of the airway changes, resulting in differences in the mechanical force or pressure applied to the airway^8–12^. In addition, age-related alterations may induce lowering of the threshold of inflammatory stimulus when the lung tissues are injured in older individuals, resulting in functional impairment^13,14^. In our study, a statistically significant difference was observed in the incidence of pneumonia between the moderate and severe groups even after adjusting for the effect of age.

The pulmonary contusion volume we measured confirmed the GGO pattern, which may include inflammatory changes and hemorrhage caused by mechanical injury or cytokines and opacity due to aspiration at the time of injury^6^. Therefore, our center initiated antibiotic treatment with ampicillin-sulbactam to prevent aspiration pneumonia when more than half of the entire lung or more than 2/3 of the unilateral lung had a contusion. Then, antibiotics were adjusted according to clinical features and sputum culture.

Pulmonary contusions are a relatively common injury in trauma patients. In addition, because a significant number of trauma patients require ventilator care in the ICU, they can be a high-risk group for respiratory complications, such as pneumonia and ARDS. Therefore, identifying these risk factors early and administering appropriate treatment, such as antibiotics, pain management, and respiratory rehabilitation, to high-risk patients is necessary. According to these data, the GCS scores, lactate levels, and the ratio of pulmonary contusion volume to the total lung volume exceeded the AUC of 0.7 in the ROC curve for predicting pneumonia. Among these, the AUC of the ratio of pulmonary contusion volume was 0.85, with the highest predictive power for future pneumonia.

This study has some limitations. First, only CT scans and history taking were performed in cases with underlying lung history. It is difficult to perform an initial pulmonary function test in trauma patients due to ICU treatment, sedation, intubation, pain, and shock. Therefore, although underlying lung history is a risk factor for future respiratory complications, it is difficult to ascertain its effect. Although the underlying lung history is identified through history taking, most patients do not know their lung function and history. Second, when chest CT is performed at the initial stage of the patient's injury, it is not performed with full inspiration. It may be difficult to view the exact volume as it is performed in various breathing phases for each patient. However, as the lung is a dynamic organ that moves continuously, we deduce that there will be difficulties in overcoming this limitation.

In conclusion, quantifying pulmonary contusion volume using initial CT in patients with chest trauma allows early identification of patients at high risk of delayed respiratory complications, such as pneumonia. Moreover, it can improve the treatment effect in patients with chest trauma by preventing complications.

## Data Availability

The datasets used and analyzed during the current study available from the corresponding author on reasonable request.
